# CELA1 Mediates Progressive Emphysema in Alpha-1 Antitrypsin Deficiency

**DOI:** 10.21203/rs.3.rs-2617812/v1

**Published:** 2023-02-23

**Authors:** Andrew J. Devine, Noah J. Smith, Rashika Joshi, Qiang Fan, Michael T. Borchers, Geremy C. Clair, Joshua N. Adkins, Brian M. Varisco

**Affiliations:** Cincinnati Children’s Hospital Medical Center; University of Cincinnati College of Medicine; Cincinnati Children’s Hospital Medical Center; Cincinnati Children’s Hospital Medical Center; University of Cincinnati College of Medicine; Pacific Northwest National Laboratory; Pacific Northwest National Laboratory; Cincinnati Children’s Hospital Medical Center

**Keywords:** Alpha-1 Antitrypsin, Emphysema, COPD, Protease

## Abstract

*Chymotrypsin-like elastase 1* (*CELA1*) is a serine protease that is neutralized by α1-antitrypsin (AAT) and prevents emphysema in a murine antisense oligonucleotide model of AAT-deficient emphysema. Mice with genetic ablation of *AAT* do not have emphysema at baseline but develop emphysema with injury and aging. We tested the role of *CELA1* in emphysema development in this genetic model of *AAT*-deficiency following tracheal lipopolysacharide (LPS), 8 months of cigarette smoke (CS) exposure, aging, and a low-dose tracheal porcine pancreatic elastase (LD-PPE) model. In this last model, we performed proteomic analysis to understand differences in lung protein composition. We were unable to show that *AAT*^−/−^ mice developed more emphysema than wild type with LPS. In the LD-PPE model, *AAT*^−/−^ mice developed progressive emphysema from which *Cela1*^−/−^&*AAT*^−/−^ mice were protected. In the CS model, *Cela1*^−/−^&*AAT*^−/−^ mice had worse emphysema than *AAT*^−/−^, and in the aging model, 72–75 week-old *Cela1*^−/−^ &*AAT*^−/−^ mice had less emphysema than *AAT*^−/−^ mice. Proteomic analysis of *AAT*^−/−^ vs. wildtype lungs in the LD-PPE model showed reduced amounts of AAT proteins and increased amounts of proteins related to Rho and Rac1 GTPases and protein oxidation. Similar analysis of *Cela1*^−/−^&*AAT*^−/−^ vs. *AAT*^−/−^ lungs showed differences in neutrophil degranulation, elastin fiber synthesis, and glutathione metabolism. Thus, *Cela1* prevents post-injury emphysema progression in *AAT*-deficiency, but it has no effect and potentially worsens emphysema in response to chronic inflammation and injury. Prior to developing anti-CELA1 therapies for AAT-deficient emphysema, an understanding of why and how CS exacerbates emphysema in *Cela1* deficiency is needed.

## Introduction

Alpha-1 antitrypsin (AAT) deficiency is a rare disease affecting approximately 1:2000 Caucasians with ~10% of these individuals developing AAT-deficient lung disease (1, 2). This lung disease is marked by progressive alveolar loss despite withdrawal of triggering agents such as cigarette smoke (3). Although the PiZZ genotype is the most common mutation, there are over 100 described variants making true population estimates difficult (4) and AAT-deficiency is typically diagnosed by reduced levels of AAT in the blood. Typically developing in the 4th and 5^th^ decades of life, AAT-deficient lung disease is marked by progressive emphysema and is the 4^th^ leading indication for lung transplantation (1). AAT augmentation therapy does not prevent disease progression (5) making the development of new therapeutic approaches critical.

*Chymotrypsin-like elastase 1* (*CELA1*) is a serine protease synthesized and secreted by alveolar type 2 cells with a physiologic role in reducing postnatal lung elastance (6). At a molecular level, CELA1 binds and cleaves non-crosslinked, hydrophobic domains of tropoelastin, and its binding to lung elastin fibers is increased with strain—similar to other pancreatic elastases (7). CELA1 is neutralized by covalent binding with AAT, and *Cela1*^−/−^ mice were completely protected from emphysema in an antisense oligonucleotide model of AAT-deficient emphysema (6). This model however did not include any injury apart from administration of the antisense oligonucleotide with levels of emphysema exceeding that seen in mice with genetic ablation of five *Serpina1* paralogues and subjected to tracheal lipopolysacharide or cigarette smoke (8). Here, we use this murine genetic model of AAT-deficiency to test the role of *CELA1* in *AAT*-deficient emphysema using multiple models to show that *CELA1* has a role in progressive airspace enlargement in AAT-deficiency that is independent of inflammation.

## Materials And Methods

### Animal Care and Use

Animal use was approved by the Cincinnati Children’s Hospital Institutional Animal Care and Use Committee (#2020–0054) with food and water provided *ad lib* with 12-hour day/night cycles. For all experiments, approximately equivalent numbers of C57BL/6 male and female mice were use. *Cela1*^−/−^ (6) and *Alpha-1 antitrypsin*-deficient (*AAT*-deficient, *Serpina1*^*em3Chmu*^/J, JAX Stock# 035015) mice were used and genotyped per published protocols (8).

### Tracheal Porcine Pancreatic Elastase Model

In 8–10-week-old mice, 0.2 to 10 units of porcine pancreatic elastase (PPE, Sigma #E1250, diluted to 20 units or 2 units per mL in PBS) was administered to mice after anesthetization with 2% isoflurane, suspension by the incisors on a rodent intubating board, tracheal cannulation with a 22-gauge angiocatheter and administration of PPE during inspiration. Mice were recovered and sacrificed at specified time intervals after intraperitoneal administration of ketamine/xylazine/acepromazine 70/5/2 mg/kg followed by exsanguination and vital organ harvest.

### Cigarette Smoke Exposure Model

Beginning at 10–12 weeks of age, mice were exposed to 4 hours of whole-body cigarette smoke five days per week for ten months using a Teague TE-10z smoking machine using smoke generated from 3R4F Kentucky Reference Cigarettes (University of Kentucky) at a concentration of 150 mg/m^3^ total suspended particulates. This work was performed under approval from Institutional Animal Care and Use Committee at the University of Cincinnati Medical Center Protocol# AM06-20-10-28-01.

### Aging Model

Male and female mice were allowed to age to 72–75 weeks and sacrificed as above.

### Lung Tissue Processing and Analysis

After exsanguination, a neck incision was made and tracheal cannula secured with 3–0 silk suture around the trachea, left bronchus ligated with a surgical clip and left lung snap frozen, and right lung inflated with 2% paraformaldehyde in PBS at 20 cm water pressure. The trachea was ligated with removal of the cannula, fixed overnight in 2% PFA, dehydrated to 100% methanol, paraffinized, and right lung lobes randomly oriented, embedded, sectioned at 5μm, and mounted. Sections were H&E stained and up to five 10X images obtained per lung lobe. Mean linear intercepts of blinded sections were determined using the methods of Dunnill (9). Airspace diameters of tile-scanned 4X sections were determined using a previously described MatLab (MathWorks, Natick, MA) code (10).

### Biochemical Assays

The upper half of snap frozen left lung lobes was homogenized in PBS and myeloperoxidase activity quantified using published protocols (11).

### Proteomic and Bioinformatic Analysis

Frozen left lung lobes were cut in half, proteins were extracted using the MPLEx methods as previously described (12). For each sample, extracted proteins were denatured, alkylated, digested with trypsin, and desalted on a C18 solid-phase extraction (SPE) cartridge (Discovery C18, 1 ml, 50 mg, Sulpelco). The peptide concentration was measured by BCA assay (ThermoScientific). Samples were analyzed using a Label free LC-MS/MS strategy. Briefly, 5 μl of samples at the concentration of 0.1 μg/μL were loaded on a SPE column and separated on a reverse phase C18 analytical column using a 180-min gradient.

MS data was recorded 60 min after injection. The effluents from the LC column were ionized by electrospray ionization by applying 2,200 V to the electrospray tip. Electrosprayed ions were introduced into the mass spectrometer (Orbitrap Q Exactive plus) via a heated capillary. The resulting ions were mass analyzed by the Orbitrap at a resolution of 70,000 covering the mass range from 300 to 1,800 Da with a maximum injection time of 20 ms and automated gain control (AGC) setting of 3e6 ions. Most abundant ions were subjected to MS2 analysis using a top12 strategy (top 12 ions are fragmented during between two precursor scans). For MS2, ions with charge states different than 1 were isolated by quadrupole mass filter in monoisotopic peak selection mode using isolation window of 1.5 Da, maximum injection time of 50 ms with AGC setting at 1e5 ions and fragmented by high-energy collision dissociation (HCD) with nitrogen at 30% normalized collision energy. Fragment ions were mass analyzed by the Orbitrap at a resolution of 17,500 covering the mass range from 200 to 2,000 Da, and spectra were recorded in the centroid mode. Ions once selected for MS2 were dynamically excluded for the next 30 seconds.

Raw mass spectrometry data searched using MaxQuant (v1.6.0.16) and LFQ data were analyzed using RomicsProcessor (13) (code DOI:10.5281/zenodo.3386527). Data were filtered for unique proteins being detected in at least 50% of samples and then log2 transformed and median normalized. Missing values were imputed using a downshifted randomly distributed data (14) ANOVA and Welch’s t-tests were performed (two-tailed, unequal variance). Protein-protein interaction networks were generated using STRING (15). Volcano plots were created using EnhancedVolcano (16).

Proteomic datasets are available at MassIVE (Accession: MSV000091330).

### Statistical Analysis

Comparisons were made using Welch’s t-test or ANOVA with Holm-Sidak *post hoc* comparison as appropriate using rstatix (17) using R-4.1.0 (18). Plots were created using ggpubr (19) with central lines indicating mean and whiskers one standard deviation. p-values of <0.05 were considered statistically significant.

## Results

### CELA1 in Tracheal Lipopolysacharide Model AAT-deficient Emphysema

**Figure 1:**
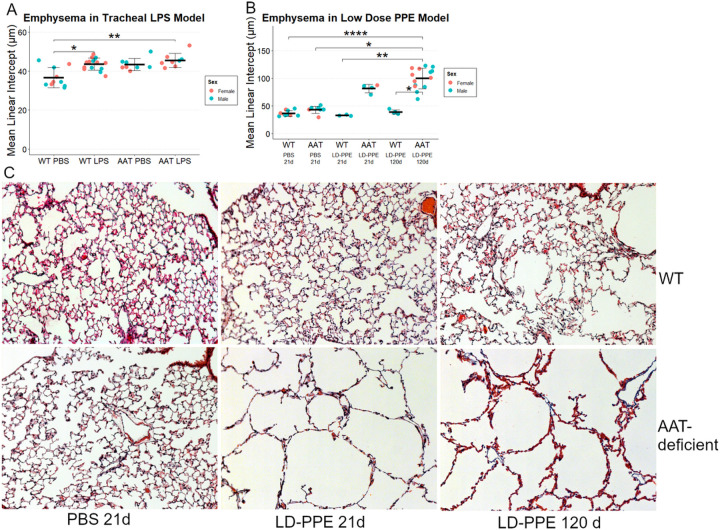
Lipopolysacharide and Low-Dose Porcine Pancreatic Elastase Models of AAT-Deficient Emphysema. (A) Wildtype (WT) mice had an increase in mean linear intercept after treatment with two doses of lipopolysaccharide (LPS) when compared to phosphate buffered saline (PBS). *AAT*-deficient mice (AAT) had some evidence of airspace simplification after PBS administration, and a level of emphysema comparable to that of WT after LPS administration. There were no clear differences by sex and comparisons are made without regard to sex. (B) WT mice did not have any significant differences in airspace size at 21 or 120 days after tracheal administration of 0.2 units porcine pancreatic elastase (LD-PPE). AAT-deficient mice had a significant increase in airspace size at both 21 and 120 days. Again, there were no clear sex-related differences and comparisons were made without regards to sex. (C) Representative 10X images of WT and AAT-deficient lungs after PBS and LD-PPE. p<0.05, **p<0.01, ****p<0.0001 by Holm-Sidak *post hoc* test.

Borel, *et al* previously reported that *AAT*-deficient mice developed more emphysema at 14 days than wildtype mice after tracheal doses of 1 and 0.5 units of lipopolysacharide at days 1 and 10 (8). We intended to test whether *Cela1*^−/−^&*AAT*-deficient mice were protected from emphysema in this model. However, we observed a similar degree of emphysema in wild-type and *AAT*-deficient mice (Figure 1A), with male and female mice similarly affected. We then performed a dose titration experiment with 1&0.5, 2&1, 5&2.5, and 10&5 units of LPS with evaluation at 21 days instead of 14 and evaluated airspace sizes using a previously described method that generates descriptive statistics of airspace sizes in tile-scanned images of all lobes (6, 10). There was no clear dose-effect and wildtype mice experienced as much or more emphysema as AAT-deficient mice (Supplemental Figure 1). Since in our hands, a previously reported LPS-based model of AAT-deficient emphysema could not be reproduced, we developed a different model of AAT-deficient emphysema.

### Low-Dose PPE Model of AAT-deficient Emphysema

Tracheal porcine pancreatic elastase (PPE) is a well-established model that causes significant injury and, in some studies, progressive emphysema (20, 21). In most studies, 1–2 units of PPE is administered tracheally, and emphysema is assessed at or before 21 days. We administered 2 units of PPE to AAT-deficient mice, and 6 of 8 mice died with extensive lung hemorrhage on necropsy. We performed a dose titration experiment and discovered that 0.2 units of tracheal PPE caused substantial emphysema in AAT-deficient mice at 21 days while wild type mice had virtually no emphysema at this dose. Furthermore, emphysema progressed over time (Figure 1B&C, Supplemental Figure 2). We therefore administered 0.2 units of tracheal PPE to AAT-deficient and *CELA1*^−/−^&*AAT*-deficient mice to test for a role for *CELA1* in emphysema in the context of AAT-deficiency.

### Lung Proteomic Changes in Low-Dose PPE Model

**Figure 2:**
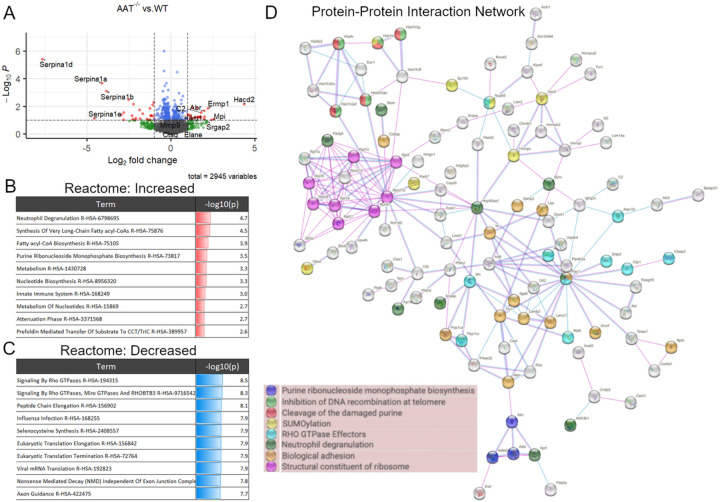
Lung Proteomics in LD-PPE model of *AAT*-deficient Emphysema. (A) A volcano plot of detected proteins 42 days after LD-PPE showed the expected low abundance of *Serpina1a*, *Serpina1b*, *Serpina1c*, and *Serpina1d* (AAT) paralogues and increased abundance of proteins related to Rho & Rac1 GTPases *Abr* and *Srgap2*, related to thrombosis and complement *Klkb1*, *C2* and *Mpi*, and related to protein oxidation *Hacd2* and *Ermp1*. Abundance of the matrix metalloproteinase *Mmp9* was increased but serine proteases neutrophil elastase (Elane) and cathepsin G (*Ctsg*) were little changed. (B) Enrichment analysis of proteins with increased abundance showed that processes related to neutrophil function, innate immunity, nucleotide metabolism, or long chaing fatty acid metabolism were increased. Scaled log10(p-values) are indicated in the right column with red indicating increased. (C) Enrichment of proteins with decreased abundance showed stronger statistical significance (blue bars) and were largely related to Rho GTPases and protein translation. (D) A protein-protein interaction network of proteins with significant (p<0.05) differences in abundance identified a number of interconnected proteins and processes with ribosomal protein synthesis, Rho-GTPases, and biological adhesion being some of the most abundant.

We performed unbiased proteomics of wild type, *AAT*-deficient, *Cela1*^−/−^ and *Cela1*^−/−^&*AAT*-deficient lungs (five per genotype) 42 days after low-dose PPE to understand the differential response to injury. In comparing wildtype and *AAT*-deficient lungs, only one protein had an adjusted p-value<0.1 and at least 2-fold change. This protein was the AAT isoform *Serpina1d* that is one of the five *AAT* paralogues deleted in this mouse(8). Seventeen genes had protein levels at least 2-fold lower in *AAT*^−/−^ compared to wildtype lungs, and 71 genes were at least 2-fold higher (Supplemental Table 1). As expected, many of the most downregulated genes in this analysis were *AAT* paralogues (e.g., *Serpina1*a*, Serpina1b,* and *Serpina1d*). Genes of many of the upregulated proteins were related to Rho & Rac1 GTPases (Abr and Srgap2), thrombosis and complement *(Klkb1, C2* and *Mpi*), and protein oxidation (*Hacd2* and *Ermp1*) (Figure 2A). Gene ontogeny enrichment for upregulated protein identified neutrophil degranulation, functions related to innate immunity, and nucleotide biosynthesis pathways as the most activated (Figure 2B). The statistical significance of downregulated pathways was greater and largely involved processes related to mRNA translation (Figure 2C). The protein-protein interaction network of both increased and decreased proteins showed many edges related to ribosomes and translation, and that many of the proteins related to neutrophil degranulation were either not adjacent in the network or not within the protein-protein interaction network at all (Figure 2D, Supplemental Figure 3). These data are consistent with the expected pulmonary biology of *AAT*-deficiency with regards to AAT synthesis and innate immunity but also some unexpected changes with regards to protein synthesis.

### CELA1 in Low-Dose PPE Model of AAT-Deficient Emphysema

**Figure 3:**
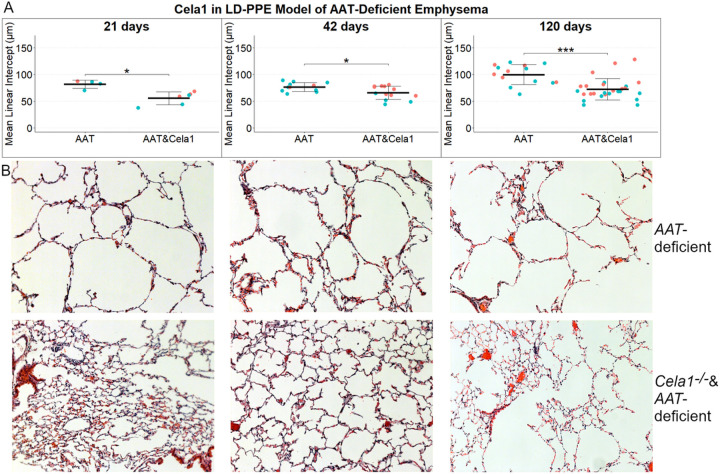
*Cela1* in the LD-PPE Model of AAT-deficient Emphysema. (A) *Cela1*^−/−^&*AAT*-deficient (AAT&Cela1) mice had less airspace destruction at 21-, 42-, and 120-days following LD-PPE compared to AAT-deficient mice. Mouse sex is not accounted for in comparisons between groups; however, female mice had more emphysema in AAT&Cela1 mice at both 42 and 120 days. (B) Representative 10X images at each timepoint. *p<0.05, ***p<0.001 by Welch’s t-test.

We tested for a role for *CELA1* in progressive emphysema by quantifying mean linear intercepts at 21, 42, and 120 days after low-dose PPE in *AAT*-deficient and *Cela1*^−/−^&*AAT*-deficient mice. Compared to *AAT*-deficient mice, *Cela1*^−/−^&*AAT*-deficient mice had less emphysema at 21, 42, and 120 days after low dose PPE administration (Figure 3A&B). Overtime, *AAT*-deficient but not *Cela1*^−/−^&*AAT*-deficient mice experienced progressive emphysema; between days 42 and 120, *AAT*-deficient mice had a 31% increase in mean linear intercept (p=0.002) while the increases in this groups between 21 and 42 days or and in the *Cela1*^−/−^&*AAT*-deficient mice over time were smaller and not significant. We observed that at 42 and 120 days, female *Cela1*^−/−^&*AAT*-deficient but not female AAT-deficient mice had more emphysema than male mice (p=0.02 and p=0.01 respectively). However, when analyzing by sex, the 120-day MLI differences remained significant for males (p=0.003) and females (p=0.03). These data indicate that *CELA1* plays a role in AAT-deficient emphysema and perhaps this role is more pronounced in males than females.

### Impact of CELA1 on Lung Proteome in Low-Dose PPE Model

**Figure 4:**
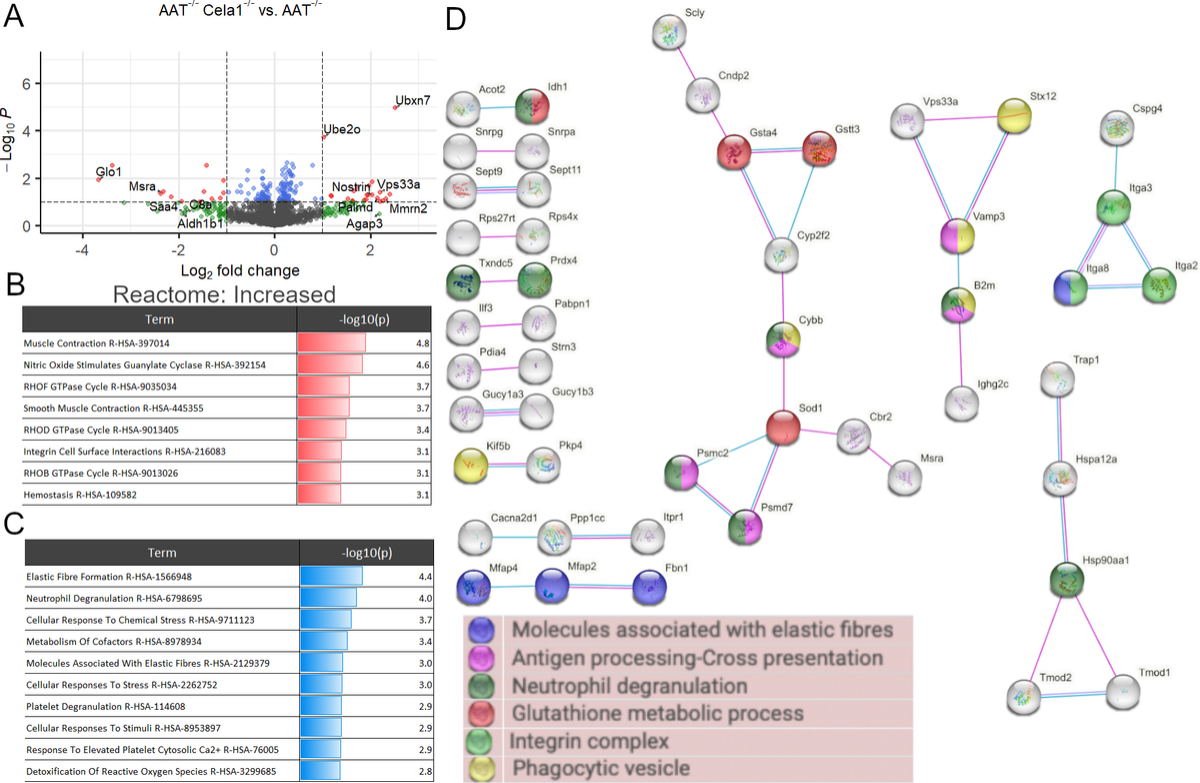
*Cela1* and the Lung Proteome of the LD-PPE model of *AAT*-deficient Emphysema. (A) A volcano plot of *Cela1*^−/−^&*AAT*^−/−^ vs. *AAT*-deficient lung 42 days after low-dose PPE showed a reduction in proteins related to oxidative stress (*Glo1*, *Msra*, and *Aldh1b1*) and acute inflammation (*Saa4* and *C8a*). Proteins related to protein homeostasis (*Ubxn7* and *Ube2o*), elastin fibers (*Mmrn2*) and related to nitric oxide and glutamic acid neuronal signaling (*Nostrin*, *Palmd*, *Agap3*, and *Vps33a*). (B) Enrichment analysis of proteins with increased abundance in *Cela1*^−/−^&*AAT*^−/−^ lungs compared to *AAT*^−/−^ lungs demonstrated increased abundance of proteins related nitric oxide and Rho GTPase signaling and processes related to muscle function. The red bar represents scaled statistical significance. (C) Analysis of proteins with decreased abundance identified differences in elastin fiber formation and neutrophil degranulation. Blue bars represent scaled statistical significance. (D) A protein-protein interaction network of proteins with significant (p<0.05) differences in abundance several smaller networks related to elastic fibers, neutrophile degranulation, and glutathione metabolism.

We then compared the lung proteomes of *Cela1*^−/−^&*AAT*-deficient and *AAT*-deficient mice 42 days after tracheal administration of low-dose PPE. There were only two proteins with adjusted p-values of less than 0.1 and at least 2-fold change. The genes of these proteins were *UBX domain-containing protein 7* and *Ig alpha chain C region*. There were 19 proteins that were at least 2-fold increased and 28 proteins that were at least 2-fold decreased in *Cela1*^−/−^&*AAT*-deficient compared to *AAT*^−/−^ mice (Supplemental Table 2). Among the proteins with increased abundance, some of the most significant were from genes related to oxidative stress (*Glo1, Msra*, and *Aldh1b1*) and acute inflammation (*Saa4* and *C8a*). Proteins that were most increased were from genes related to ubiquitination (*Ubxn7* and *Ube2o*), elastin fibers (*Mmrn2*), and neurotransmitters (*Nostrin*, *Palmd*, *Agap3*, and *Vps33a*) (Figure 4A). Gene ontogeny enrichment for proteins that were increased showed abundance of Rho GTPase proteins, nitric oxide signaling, and muscle-related proteins (Figure 4B). Downregulated processes included elastic fiber formation and neutrophil degranulation (Figure 4C). Protein-protein interaction analysis of increased and decreased proteins identified a number of small but connected networks related to elastin fibers, neutrophil degranulation, and glutathione metabolism (Figure 4D). These data demonstrate that *Cela1* mediates protein-level differences in elastin microfibril associated proteins in this AAT-deficient model of emphysema and that there are also potential differences in neutrophil activity and oxidative state.

### CELA1 in Cigarette Smoke Induced Emphysema

**Figure 5:**
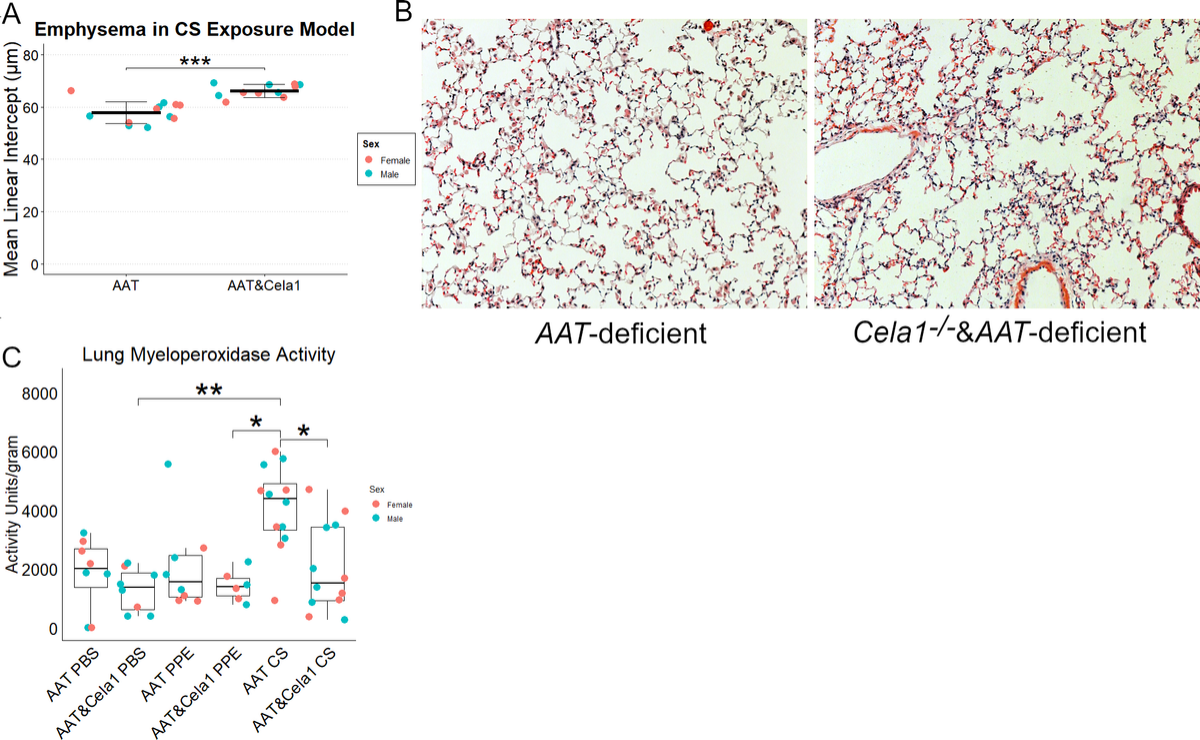
Cela1 and CS exposure in AAT-deficiency. (A) After 10 months of whole-body cigarette smoke exposure, *Cela1*^−/−^&*AAT*-deficient mice had more airspace simplification than *AAT*-deficient mice. Sex is shown for comparison only and was not used in the shown comparison. ***p<0.001 by Welch’s t-test. (B) representative H&E images of CS-exposed mouse lung. (C) Lung myeloperoxidase activity was greater in *AAT*-deficient mice exposed to CS compared to *Cela1*^−/−^&*AAT*-deficient mice and at 42 days post-PBS or PPE in both genotypes *p<0.05 **p<0.05 by Dunn’s post hoc test. Wilcoxon rank sum p<0.01.

As cigarette smoke exposure is highly associated with the development of emphysema in AAT-deficient individuals, we performed whole body cigarette smoke exposure to *AAT*-deficient and *Cela1*^−/−^&*AAT*-deficient mice five days a week for 10 months. Contrary to our expectations, and *Cela1*^−/−^&*AAT*-deficient mice were not protected from CS-induced emphysema and *Cela1*^−/−^&*AAT*-deficient mice had more evidence of airspace destruction than *AAT*-deficient mice (Figure 5A&B). Given the proteomic findings of altered neutrophil associated and chemotaxis proteins in *Cela1*^−/−^&*AAT*-deficient mice, we hypothesized neutrophil activity might be increased. However, myeloperoxidase activity was reduced by more than half in *Cela1*^−/−^&*AAT*-deficient mice compared to *AAT*-deficient mice in response to CS and comparable to activity levels seen 42 days after PPE and PBS. (Figure 5C). While the mechanism is unclear, these data indicate the absence of *Cela1* enhances airspace destruction in *AAT*-deficient mice response to sustained cigarette smoke exposure.

### CELA1 in Age-Related Alveolar Simplification

**Figure 6:**
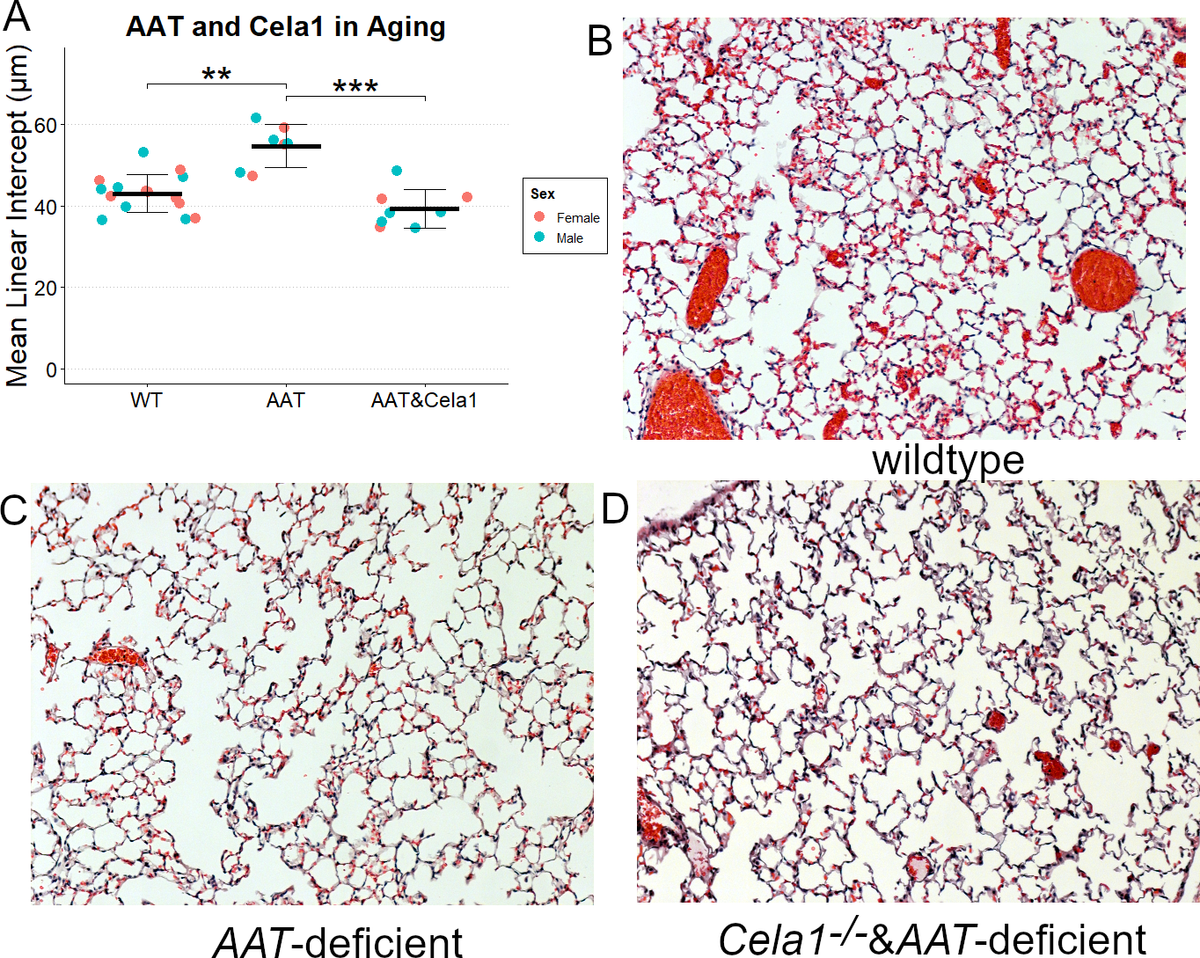
*Cela1* in Age-related Alveolar Simplification in AAT-deficiency. (A) At 72–75 weeks of age, *Cela1*^−/−^&*AAT*-deficient mice had a degree of alveolar simplification that was comparable to that of wildtype (WT) mice. Sex is shown for comparison purposes and was not included in analysis. (B) Representative 10X photomicrographs of wildtype (C) AAT-deficient, and (D) *Cela1*^−/−^&*AAT*-deficient mice.

In humans and mice, aging is associated with slowly progressive airspace simplification, and AAT-deficient mice were reported to have more rapid simplification that wildtype (8). We validated these findings and discovered that *Cela1*^−/−^&*AAT*-deficient mice experienced a level of age-related airspace simplification similar to that of wildtype mice (Figure 6).

## Discussion

This is the first study to show in a novel mouse model of injury-induced emphysema in AAT-deficiency, that *Cela1* is required for progressive airspace enlargement, and that this gene is important in age related alveolar simplification. Interestingly, *Cela1* did not play a role in cigarette smoke-induced emphysema suggesting that the mechanism by which chronic inflammation and injury causes emphysema is independent of the post-injury remodeling program. When considered in the context of our previous work showing that *Cela1* binds to lung elastin in the context of strain (6), these findings support the concept that altered local lung mechanics in emphysema promote CELA1-mediated remodeling leading to progressive emphysema and that the lack of neutralization of Cela1 protein by AAT exacerbates this process.

The four models used in our studies help elucidate the role of CELA1 in AAT-deficient emphysema. In our first experiment, we were unable to recapitulate the emphysema seen with LPS administration in *AAT*-deficient mice (8). Potential reasons for this could be differences in microbiome, reduced LPS potency, or differences in administration technique. The finding that increasing doses of LPS caused more airspace injury in wildtype than *AAT*-deficient mice supports the conclusion that some intrinsic difference between facilities is responsible—a hypothesis that could lead to improved understanding of emphysema pathogenesis if followed up upon. For our second experiment, we modified the tracheal PPE model for use in *AAT*-deficient mice. The observed pulmonary hemorrhage and lethality with standard dose of PPE in *AAT*-deficient mice is probably because after tracheal PPE administration, there is leakage of serum into the airspace allowing AAT to neutralize of the proteases in PPE. In *AAT*-deficient mice, the proteases in PPE remain active for a longer period leading to more extensive damage and death. Low-dose PPE administration to *AAT*-deficient mice produced robust emphysema that was progressive—an important clinical feature of AAT-deficient emphysema. The cigarette smoke exposure model is more clinically relevant than the PPE model, but it is dissimilar to human AAT-related lung disease because while smoking cessation is a mainstay of treatment for human AAT-related lung disease, in the mouse model, exposure is continuous. Given the relatively small increase in MLI observed, cessation of cigarette smoke exposure experiments would need a large number of mice and are probably inappropriate for *Cela1* studies since some consequence of its absence seems to worsen CS-induced emphysema. Notably, Steams *et al* recently showed that AAT knockdown using antisense oligonucleotides increased *Cela1* expression but did not worsen lung injury in a cigarette smoke and influenza model (22). Lastly, we validated previously reported findings regarding age-related alveolar simplification in AAT-deficient mice (8) and showed that *CELA1* plays an important role in this process. In considering these findings in the context of the physiologic role of *CELA1*—namely the reduction of postnatal lung elastance (6)—data from these four models demonstrate that in the context of AAT-deficiency, *CELA1* does not appear to be involved in the lung’s response to injury but is important for continued airspace destruction once emphysema is established.

This study adds to our understanding on the role of sex on AAT-deficient emphysema. We previously reported that in the antisense oligonucleotide model, AAT-deficient mice had more significant emphysema (23), but we did not see these same changes in the genetic model. In the current study, while *Cela1* ablation reduced emphysema in both male and female *AAT*-deficient mice in the LD-PPE model, the protection was greater in male mice. Previous studies did not show any effect of sex on lung *Cela1* expression (6). These findings suggest that *CELA1* could play a role in the increased sensitivity of AAT-deficient female patients compared to male ones (24).

The progressive nature of emphysema in mouse models controversial. In mice, rats, and rabbits studies show that emphysema improves (25), is stable (26–28), or progresses (20, 21, 29) over time. While comparison between studies of different species and doses of PPE are difficult, it does appear that studies with stable or improved MLI have lesser degrees of initial injury than those that show progression. The role of localized lung tissue mechanics in emphysema progression was convincingly demonstrated by Toumpanakis *et al*. Mice treated with PPE were followed to 21 days with subsequent reduction of tracheal cross-sectional area by 50% for 24 or 72 hours. Emphysema was substantially greater with 72 hours of airflow restriction (30). Perhaps there is threshold of alveolar injury beyond which localized tissue mechanics become too deranged to make re-alveolarization possible; a hypothesis that warrants future study as understanding key processes of repair-regeneration in emphysema could lead to novel therapies to restore lung function in emphysema. There is likely an age-related component to repair potential as well since older mice have less compensatory lung growth than younger ones (31). While in human emphysema, alveolar loss is considered permanent, adult human lung has the potential to re-initiate alveolarization post-pneumonectomy (32) lending hope to the idea that if emphysema progression could be halted, other therapies could be employed to regenerate damaged lung tissue.

Our study has several limitations. First, we did not determine if *Cela1*^−/−^&*AAT*-deficient mice had preservation of lung function or diffusing capacity, and we did not measure lung volume in a way that would have permitted calculation of alveolar surface area. Our aged mice were harvested at 15 months and not 18 or 24 months as in some studies. We decided on this time point after an unexpected mortality in an aged AAT-deficient mice at 15 months and concern for losing additional mice. To the best of our knowledge, there are no reports of early mortality in *AAT*-deficient mice. We previously identified *CELA1* as being expressed in alveolar type 2 cells, but we did not provide any cell-specific information here. Lastly, while we showed a specific role for CELA1 in this process, we did not interrogate the role of other proteases or test whether AAT-replacement was sufficient to protect mice from emphysema in the various models tested. As AAT has anti-inflammatory properties (33), it may be that CELA1 inhibition and AAT replacement therapy could be complementary.

## Conclusions

CELA1 mediates progressive airspace destruction in AAT-deficient emphysema and its inhibition may represent a novel therapy in AAT-related lung disease.
